# *In vivo* X-Nuclear MRS Imaging Methods for Quantitative Assessment of Neuroenergetic Biomarkers in Studying Brain Function and Aging

**DOI:** 10.3389/fnagi.2018.00394

**Published:** 2018-11-27

**Authors:** Xiao-Hong Zhu, Wei Chen

**Affiliations:** Center for Magnetic Resonance Research (CMRR), Department of Radiology, School of Medicine, University of Minnesota, Minneapolis, MN, United States

**Keywords:** *in vivo* X-nuclear MRS imaging, brain energy metabolism, neuroenergetics, mitochondrial function, ultra-high magnetic field (UHF), aging, neurodegeneration

## Abstract

Brain relies on glucose and oxygen metabolisms to generate biochemical energy in the form of adenosine triphosphate (ATP) for supporting electrophysiological activities and neural signaling under resting or working state. Aging is associated with declined mitochondrial functionality and decreased cerebral energy metabolism, and thus, is a major risk factor in developing neurodegenerative diseases including Alzheimer’s disease (AD). However, there is an unmet need in the development of novel neuroimaging tools and sensitive biomarkers for detecting abnormal energy metabolism and impaired mitochondrial function, especially in an early stage of the neurodegenerative diseases. Recent advancements in developing multimodal high-field *in vivo* X-nuclear (e.g., ^2^H, ^17^O and ^31^P) MRS imaging techniques have shown promise for quantitative and noninvasive measurement of fundamental cerebral metabolic rates of glucose and oxygen consumption, ATP production as well as nicotinamide adenine dinucleotide (NAD) redox state in preclinical animal and human brains. These metabolic neuroimaging measurements could provide new insights and quantitative bioenergetic markers associated with aging processing and neurodegeneration and can therefore be employed to monitor disease progression and/or determine effectiveness of therapeutic intervention.

## Mitochondrial Dysfunction and Neuroenergetic Deficiency as Hallmarks of Aging and Neurodegeneration

Aging is an inevitable process of life. With the rapid growth of the elderly population, brain diseases associated with functional decline and neurodegeneration, such as cognitive impairment (CI), Alzheimer’s disease (AD) and Parkinson’s disease (PD), not only have a huge impact on people’s quality of life, but also greatly increase the social and economic burdens. As a complex biological process, aging (defined as an age-progressive decline in intrinsic physiological function) can be influenced by many factors other than the actual age, such as heredity, lifestyle, income and living environment.

Mitochondria are organelles found in the cells of complex organism and they produce >90% of the adenosine triphosphate (ATP) energy molecules in the brain via the oxidative phosphorylation of adenosine diphosphate (ADP). In addition to supporting unceasing neuronal activity, neurotransmission, cellular signaling and other functions under different brain states, approximately one-quarter of total ATP energy expenditure in the human brain is used for biosynthesis and “housekeeping” functions to maintain cellular integrity (Siesjo, 1978; Erecińska and Silver, 1989; Barinaga, 1997; Rolfe and Brown, 1997; Boyer, 1999; Attwell and Laughlin, 2001; Shulman et al., 2004; Hyder et al., 2006; Du et al., 2008; Zhu et al., 2015a). A coupling relationship between the neuronal activity and ATP energy consumption of the brain tissue holds for a wide range of physiological conditions and brain relies on an effective metabolic regulation to balance the ATP supply and demand through key biochemical reactions associated with energy metabolism (Du et al., 2008; Zhu et al., 2012, 2018). Under normal circumstances, the mitochondrial ATP production rate in the brain is indirectly but closely coupled with the cerebral metabolic rates of glucose (CMR_Glc_) and oxygen (CMRO_2_) and tightly regulated by the nicotinamide adenine dinucleotide (NAD) redox state, which can be determined by the intracellular concentration ratio of the oxidized (NAD^+^) and reduced (NADH) NAD molecules (i.e., NAD redox ratio: RX_NAD_).

**Figure 1:**
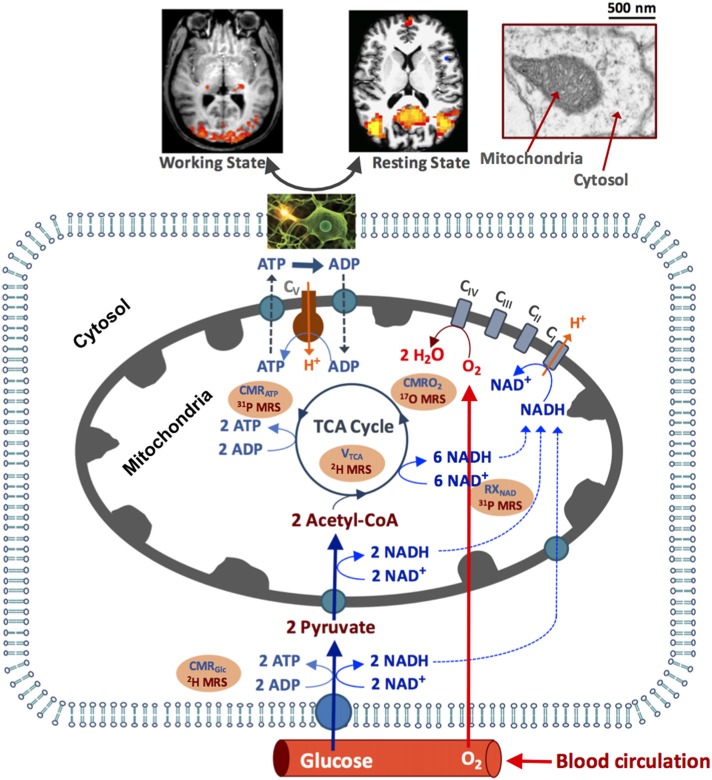
Schematic illustration of major brain hemodynamic and metabolic processes occur in capillary, cytosol and mitochondrial sub-cellular compartments. The feeding arteries supply oxygen and glucose to the cell; glucose is converted to pyruvate, which enters mitochondrial tricarboxylic acid (TCA) cycle and oxidative metabolism pathways. The oxygen utilization is generally coupled with the adenosine triphosphate (ATP) production via the oxidative phosphorylation of adenosine diphosphate (ADP). The ATP is utilized in cytosol to support electrophysiological activities and brain functions at resting and/or working state. The NAD redox reactions are essential in regulating the ATP energy production. The cerebral metabolic rates of glucose (CMR_Glc_), oxygen (CMRO_2_) and ATP production (CMR_ATP_), TCA cycle rate (V_TAC_) and nicotinamide adenine dinucleotide (NAD) redox ratio (RX_NAD_) representing the metabolic activities of the brain tissue can be noninvasively measured or imaged using the advanced *in vivo* X-nuclear ^2^H, ^17^O and ^31^P MRS approaches as depicted in the shadowed texts with orange background. C_I_-C_V_ represent five enzyme complexes involving in the cellular respiration chain reactions.

As depicted in Figure 1, the circulating blood flow constantly supplies oxygen and glucose to the brain tissue, where the glucose is transported into the brain cells and converted to pyruvate via glycolysis and produces two ATP and two NADH molecules from each glucose molecule consumed in the cytosol. Most pyruvate molecules enter the mitochondria to form acetyl Co-A, its oxidation via the tricarboxylic acid (TCA) cycle produces eight NADH molecules that can be converted to NAD^+^ molecules through oxygen metabolism (Stryer, 1988). The electron transport chain reactions extrude H^+^ ions from mitochondria to generate an electrochemical potential gradient across the mitochondrial inner membrane, which is the driving force for the mitochondrial F_1_F_0_-ATP_ase_ mediated enzyme reaction that synthesizes ATP from ADP and inorganic phosphate (Pi; producing >30 ATPs per consumed glucose under physiological condition) and transports the H^+^ ions back into mitochondria (Siesjo, 1978; Hyder et al., 2006). The ATP utilization occurs in the cytosol via the ATP hydrolysis reaction. The majority of the ATP energy are used to maintain the Na^+^/K^+^ ion gradients across the cell membrane for supporting action potential propagation, neuronal firing and neurotransmitter cycling (Siesjo, 1978; Stryer, 1988; Shulman et al., 1999). The rapid ATP turnover requires efficient transportation of the ATP molecules between the cytosolic and mitochondrial compartments to maintain the intracellular ATP homeostasis. This is accomplished partly by a creatine kinase (CK) catalyzed near-equilibrium chemical exchange between ATP + Creatine (Cr) and phosphocreatine (PCr) + ADP (Kemp, 2000; Du et al., 2008).

Although it only accounts for 2% of the total body weight, the human brain has enormous energy needs. The resting adult brain receives approximately 15% of the cardiac output and uses most (~20%) of systemic oxygen and glucose consumptions (Raichle, 1987; Shulman et al., 2004; Hyder et al., 2006). It is worth noting that the intracellular ATP concentration in the brain is very low (~3 mM); the entire human brain only contains approximately 2 g of ATP (assuming an average adult brain weight of 1.4 kg). In contrast, the rate of ATP synthesis by the F_1_F_0_-ATP_ase_ reaction is very high (8–9 μmole/g/min) in the human brain (Lei et al., 2003a; Du et al., 2007; Zhu et al., 2012), indicating that a human brain produces 7–8 kg of ATP molecules (about 5–6 times of the human brain weight) in 1 day. The extreme high turnover between the ATP production and utilization is critical in fulfilling the high energy demand of the neuronal cells and maintaining the intracellular ATP homeostasis.

The conversion between NAD^+^ and NADH through the NAD redox reaction determines the intracellular NAD redox state, which controls the balance of cytosolic glycolysis and mitochondrial oxidative phosphorylation to produce adequate ATP molecules (Chance et al., 1962; Lu et al., 2014b; Zhu et al., 2015b). Mitochondrial dysfunction and energy deficiency are the key cellular hallmarks of aging and neurodegeneration, suggesting that mitochondria can serve as therapeutic targets for various neurodegenerative diseases or for monitoring the aging processes (Creasey and Rapoport, 1985; Rapoport, 1999; Balaban et al., 2005; Guarente, 2008; Yap et al., 2009; Reddy and Reddy, 2011; Nunnari and Suomalainen, 2012; López-Otín et al., 2013; Pathak et al., 2013; Lin et al., 2014; Yin et al., 2014). Therefore, the development of quantitative, reliable and sensitive neuroimaging tools or biomarkers capable of assessing mitochondrial function and cerebral energy metabolism is essential for studying the underlying mechanisms of human brain aging and monitoring the progression of aging-related brain disorders. Furthermore, biomarkers with improved specificity and sensitivity can be potentially used to distinguish normal aging from neurodegeneration, provide early diagnoses, identify therapeutic targets and evaluate treatment efficacy.

## Development of Neuroimaging Biomarkers for Studying Aging and Underlying Mechanism in Human Brain

Modern neuroimaging techniques have played important roles in study of human brain aging and diagnosis of neurodegenerative diseases; in particular, Positron Emission Tomography (PET) has been well established to evaluate regional brain glucose and oxygen utilization, neurochemical and neurotransmitter changes, and inflammation in AD and PD brains (Borghammer et al., 2010; Brooks and Pavese, 2011; Niccolini et al., 2014; Varley et al., 2015). For instance, the PET imaging based on radioactive fludeoxyglucose (^18^FDG) is used to measure the glucose uptake rate that thought to reflect CMR_Glc_. The ^18^FDG-PET has been extensively employed to study human brain aging; however, contradictory findings with either negative (Duara et al., 1983, 1984) or positive (Pantano et al., 1984; Yamaguchi et al., 1986; Leenders et al., 1990; Marchal et al., 1992; De Santi et al., 1995; Goyal et al., 2017) correlation between actual age and CMR_Glc_ in healthy human have been reported. Note that ^18^FDG-PET based CMR_Glc_ image reflects the total glucose metabolism through both mitochondrial oxidative phosphorylation and aerobic glycolysis pathways including the conversion of pyruvates (products of glycolysis) into lactates; therefore, it does not directly represent the actual mitochondrial neuroenergetics, which can be determined by CMRO_2_. Significant CMRO_2_ reductions in elderly people have been reported, indicating a tight correlation between the mitochondrial energy metabolism and aging (Yamaguchi et al., 1986; Leenders et al., 1990). The CMRO_2_ decline is consistent with significant decreases in respiratory enzyme (Complexes I–V) activities observed in aging mice brain (Ferrándiz et al., 1994; Navarro and Boveris, 2007), and is in line with a human brain study showing an approximately 30% reduction in both neuronal oxidative glucose metabolism and neurotransmission cycling rates in elderly people (Boumezbeur et al., 2010).

However, there is a lack of sophisticated neuroimaging methods that can quantitatively and noninvasively assess brain mitochondrial enzymatic activities and ATP bioenergetics, even though they play a central role in human aging and neurodegeneration. Most predictive biomarkers offered by neuroimaging are neither sufficiently nor proximal to sub-cellular mechanisms of aging to link mitochondrial and ATP bioenergetic functions. In this article, we will provide a brief review of several advanced metabolic neuroimaging methods that are based on *in vivo* X-nuclear magnetic resonance (MR) spectroscopic (MRS) imaging (MRSI) at ultra-high magnetic field (UHF) for noninvasive imaging and quantitative assessment of human brain mitochondrial functions and associated bioenergetic biomarkers, which could be highly sensitive to aging without using radioactive tracers. Three *in vivo* X-nuclear MRS methods for imaging cerebral energy metabolisms following specific metabolic pathways are discussed:

1. *In vivo* deuterium-2 (^2^H) MRSI method for simultaneously measuring CMR_Glc_ and the TCA cycle rate (V_TCA_),
2. *In vivo* oxygen-17 (^17^O) MRSI method for quantitatively imaging three physiological parameters: CMRO_2_, cerebral blood flow (CBF) and oxygen extraction fraction (OEF),
3. *In vivo* phosphorus-31 (^31^P) MRSI method for simultaneous measurement of cerebral metabolic rates of ATP synthesis via the ATP_ase_ reaction (CMR_ATP_) and CK reaction (CMR_CK_), as well as for measuring intracellular NAD^+^ and NADH, thus, the NAD redox ratio (RX_NAD_).

These X-nuclear MRSI methods provide complementary measurements of brain energy metabolisms and ATP bioenergetics following the metabolic roadmap as shown in Figure 1.

*In vivo* carbon-13 (^13^C) MRS is another X-nuclear MRS method that has been used to study energy metabolism and neurotransmission in animal and human brains. By combining dynamic ^13^C MRS with ^13^C-labeled substrates administration and compartmentalized quantification model, the metabolic fluxes of various pathways involving glucose metabolism and neuronal-astrocyte compartmental exchange can be assessed via monitoring the ^13^C-label incorporation to the major metabolites along these pathways. The strength and limitations of the ^13^C MRS technique and its applications have been extensively reviewed (e.g., Rothman et al., 2011 #1543; Rodrigues et al., 2013 #1544; Sonnay et al., 2017 #1542), and thus, is covered in this article.

## Limitations of *in vivo* X-Nuclear MRSI and Advantages of Ultra-High Field

To apply the *in vivo* X-nuclear MRS or MRSI in biomedical research, we face many challenges, in particular, owing to the very low concentration of detectable metabolites (in the range of few or sub-millimolar (mM)) that is several to tens of thousands of times lower than the tissue water content detected by ^1^H MRI. Additionally, since the gyromagnetic ratios of the X-nuclei (e.g., ^2^H, ^13^C, ^17^O and ^31^P) are several times lower than that of ^1^H, the intrinsic detection sensitivity and signal-to-noise ratio (SNR) of the X-nuclear MRS are further reduced, thus, extensive signal averaging is required to achieve reasonable SNR and spatial resolution. These factors have limited the reliability, applicability and spatiotemporal resolution of the *in vivo* MRSI measurements. To address these limitations, it has been shown that UHF scanners can provide a significant SNR gain and improve spectral and spatial resolutions. The advantages of the UHF for *in vivo* ^31^P and ^17^O MRS brain applications are described below.

The ^31^P nuclide has been studied extensively since the inception of *in vivo* MRS (Shulman et al., 1979; Ackerman et al., 1980; Shoubridge et al., 1982). Besides high energy phosphate compounds (ATP and PCr) and Pi, other phosphorus metabolites such as NAD^+^ and NADH that are actively involved in the NAD redox reaction, glycerophosphoethanolamine (GPE), glycerophosphocholine (GPC), phosphoethanolamine (PE) and phosphocholine (PC) which are essential to membrane phospholipid metabolism could also be detected by *in vivo* ^31^P MRS. The reduced resonance linewidths (in the ppm unit) at higher field will significantly improve the ^31^P spectral resolution, which makes it possible to resolve adjacent or overlapped phosphate resonances, determine the redox ratio of NAD (Lu et al., 2014b, 2016a; Zhu et al., 2015b), and distinguish intracellular and extracellular Pi *in vivo*. Interestingly, the T_1_ values of most phosphorus metabolites decrease at higher fields, presumably the chemical shift anisotropy (CSA) dominates the longitudinal relaxation mechanism at UHF. The shortened T_1_ allows more signal averaging per unit sampling time, thus, further improves the SNR and leads to a super linear dependence of the ^31^P MRS sensitivity on the magnetic field strength (B_0_) after considering the B_0_ dependences of T_1_ and resonance linewidth (Qiao et al., 2006; Lu et al., 2014a).

^17^O is a stable and NMR detectable isotope of oxygen; it has a very low natural abundance (0.037%) and one-seventh gyromagnetic ratio of the ^1^H. The ^17^O isotope with a quantum number of 5/2 obeys the quadrupolar relaxation mechanism, thus, the ^17^O nuclide in water (H_2_^17^O) has very short longitudinal (T_1_) and transverse (T_2_, or apparent T_2_: ${\text{T}}_{2}^{*}$) relaxation times (<7 ms) that are insensitive to the B_0_ inhomogeneity (Zhu et al., 2001, 2005; Lu et al., 2013). The SNR of the ^17^O brain water signal has an approximate quadratic field dependence on the static magnetic field strength (i.e., SNR ∝ B_0_^2^; Zhu et al., 2001; Lu et al., 2013), while the ^1^H MRI has an approximate linear field dependence (Vaughan et al., 2001). The field dependence of the brain H_2_^17^O signal across a wide range of B_0_ indicates an over 120 times SNR gain at 16.4T as compared to a 1.5T clinical MRI scanner. Therefore, it is possible to obtain three-dimensional (3D) ^17^O MRSI of the animal or human brain with adequate SNR and reasonable spatiotemporal resolution at ultrahigh fields. Furthermore, the sensitivity gain at UHF is essential for the development of the *in vivo* ^17^O MR-based neuroimaging methodology in assessing cerebral oxygen metabolism and perfusion. The UHF advantages are also expected in *in vivo* ^2^H MRSI applications owing to a similar quadrupolar relaxation mechanism.

## Simultaneous Assessment of CMR_Glc_ and V_TCA_ Using *in vivo* ^2^H MRS Technique

CMR_Glc_ and V_TCA_ are key parameters presenting the rates of glucose metabolism in brain tissue. Ability to quantify their values *in vivo* is crucial for assessing the metabolic and energetic states of the brain. As shown in Figure 1, the stoichiometric ratio of the CMR_Glc_ and V_TCA_ in normal brain is approximately two to one since one glucose can produce two pyruvates in cytosol before entering the mitochondrial TCA cycle; such coupling relationship can change under pathological condition, e.g., in brain tumor or stroke. Even though it is challenging, quantitative and simultaneous imaging of both CMR_Glc_ and V_TCA_ is desired for studying the complex glucose metabolic pathways and their contributions to the ATP production under normal and diseased states. Recently, we have developed an *in vivo* ^2^H MRS technique for simultaneous CMR_Glc_ and V_TCA_measurement; this technique has been validated at 16.4T using a preclinical rat model (Lu et al., 2017).

^2^H nuclide is a stable isotope of hydrogen with a quantum number of 1 and has an extremely low natural abundance (0.0156%). Like ^17^O nuclide, molecules containing ^2^H obey quadrupolar relaxation mechanism and have short T_1_ and T_2_ values that enables rapid signal averaging for gaining the SNR. Thus, the *in vivo* ^2^H MRS or MRSI becomes attractive at UHF when combining with ^2^H-isotope (deuterium) labeled glucose infusion (Mateescu et al., 2011; Lu et al., 2017). After infusion, several deuterium labeled compounds, including the glycolysis and TCA cycle intermediates of the brain tissue, e.g., glutamate/glutamine (Glx), lactate (Lac) and water, can be detected using the UHF ^2^H MRS with excellent sensitivity and temporal resolution and identified based on their well-resolved ^2^H resonances and chemical shifts. The robust ^2^H MRS signal detection, spectral analysis and kinetic modeling eventually allow for quantification of CMR_Glc_ and V_TCA_ in live brains.

**Figure 2:**
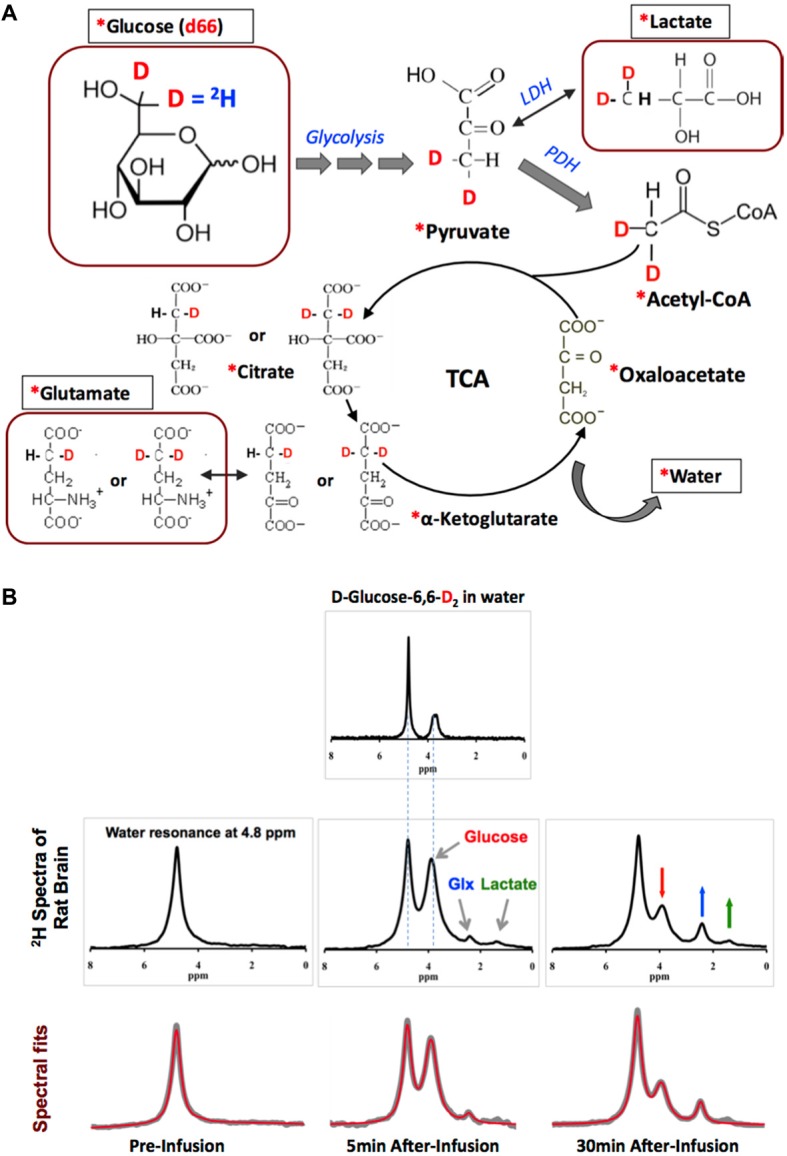
**(A)** The ^2^H-labeling scheme following the metabolic pathways of isotope-labeled glucose D-Glucose-6,6-d_2_ (d66). The ^2^H-labeled glucose first incorporates into pyruvate pool through glycolysis to form [3,3-d_2_] pyruvate, some of which can be converted to [3,3-d_2_] lactate by lactate dehydrogenase (LDH). [3,3-d_2_] Pyruvate can also be transported into the mitochondria to form [2,2-d_2_] Acetyl-CoA catalyzed by pyruvate dehydrogenase (PDH). After entering the TCA cycle, intermediates (4-d) or [4,4-d_2_] citrate and (4-d) or [4,4-d_2_] α-ketoglutarate could exchange with glutamate to generate (4-d) or [4,4-d_2_] glutamate. In this process, the ^2^H-labels may exchange with the proton(s) in water molecule to form deuterated water and depart from the cycle. “*”: Pools labeled with ^2^H; square boxes: highlighting the metabolites detectable by *in vivo* ^2^H MRS. **(B)** Representative original (upper rows black traces and bottom row gray traces) and fitted (red traces in bottom row) ^2^H spectra obtained from deuterated glucose (d66) phantom solution (top panel), and in rat brain pre- (left column) and 5 or 30 min post-deuterated glucose (d66) infusion. ^2^H resonance assignments: water at 4.8 ppm (use as a chemical shift reference); glucose at 3.8 ppm; mixed glutamate and glutamine (Glx) at 2.4 ppm; and lactate at 1.4 ppm. Figure adapted from Lu et al. (2017).

Figure 2A displays the ^2^H-isotope labeling scheme, labeled metabolites and associated metabolic pathways following an intravenous D-Glucose-6,6-d_2_ (d66) infusion (Mateescu et al., 2011; Lu et al., 2017), where d66 glucose and non-labeled glucose are transported together into the brain and metabolized via glycolysis and oxidative phosphorylation. Along the metabolic pathways, the deuterium label on d66 can incorporate into the Lac, Glx and water pools, which can then be monitored through dynamic ^2^H MRS acquisitions. Excellent spectral quality and spectral fittings can be obtained not only from d66 phantom solution (with water resonance set at 4.8 ppm as a chemical shift reference) but also from living rat brain; for instance, well-resolved deuterated resonances of glucose (3.8 ppm), Glx (2.4 ppm) and lactate (1.4 ppm) were detected following a brief (2 min) d66 infusion (Figure 2B). Their dynamic signal changes (15 s temporal resolution) were used to determine the CMR_Glc_ and V_TCA_ values based on a simplified kinetic model (Lu et al., 2017). The *in vivo* ^2^H MRS approach has been applied to rat brains under isoflurane anesthesia and morphine analgesic condition; significant reduction of CMR_Glc_ and V_TCA_ in rat brains under 2% isoflurane (CMR_Glc_ = 0.28 ± 0.13 and V_TCA_ = 0.6 ± 0.2 μmol/g/min) as compared to that of morphine (CMR_Glc_ = 0.46 ± 0.06 and V_TCA_ = 0.96 ± 0.4 μmol/g/min) were found (Lu et al., 2017), suggesting that the *in vivo* ^2^H MRS technique is highly sensitive in detecting the cerebral metabolic rate changes.

Compared with the *in vivo* ^13^C MRS (Gruetter et al., 2003), several merits of the *in vivo* ^2^H MRS technology are worth mentioning: (i) the short T_1_ relaxation time of the quadrupolar ^2^H nuclide (e.g., ~50 ms for d66 in rat brain at 16.4T; Lu et al., 2017) enables rapid sampling to significantly increase the SNR for *in vivo* ^2^H MRS or MRSI application (see an example in Figure 2B); (ii) the chemical shift assignments (in ppm) and spectral patterns of the deuterated metabolites are almost identical to that of *in vivo* ^1^H MRS, while the chemical shift range (in Hz) of the ^2^H spectrum is ~7 times narrower than that of ^1^H MRS due to a much lower ^2^H gyromagnetic ratio (6.5 MHz/T for ^2^H vs. 42.6 MHz/T for ^1^H), thus, the chemical shift displacement artifacts should be significantly reduced for ^2^H MRS localization, especially at UHF (Chen and Zhu, 2005; Lu et al., 2017); on the other hand, it is challenging to study the neurotransmission cycling between neuron and glia cells using the ^2^H MRS method due to the inability of resolving ^2^H-labeled glutamate from glutamine (Sibson et al., 2001; Hyder et al., 2006); (iii) in an *in vivo* ^2^H MRS spectrum, the natural abundant water signal of the brain tissue can serve as an internal reference for quantifying cerebral metabolites labeled with deuterium, which makes metabolites quantification easier and more reliable; and (iv) there is no background contamination in the ^2^H spectrum of living brain because no natural abundance metabolite signal other than water is detectable *in vivo*, therefore, technique commonly applied in ^13^C and ^1^H MRS to suppress intense water or lipid signal is no longer needed.

The *in vivo* ^2^H MRS approach could be highly valuable for studying the decoupled relationship between glycolysis and oxidative metabolism and image the Warburg effect in brain tumor. This can be achieved through directly measuring the metabolic rates of CMR_Glc_ and V_TCA_ using the ^2^H MRSI approach or by simply mapping the Glx/Lac ratio (ideally measured when Glx and Lac signals reaching a plateau after the introduction of d66), which could provide a sensitive index of the Warburg effect in brain tumor (Lu et al., 2016b). To establish a completely noninvasive metabolic imaging based on the *in vivo* ^2^H MRSI measurement, the intravenous infusion of the d66 tracer can be replaced by an oral delivery of d66. The feasibility of introducing d66 via oral intake for CMR_Glc_ and V_TCA_ measurement has been recently demonstrated (Lu et al., 2018), which paves the way for translational application.

## Non-invasive Imaging of CMRO_2_, CBF and OEF Using *in vivo* ^17^O MR Technique

The motivation of developing *in vivo* ^17^O MR imaging techniques is to measure CMRO_2_ via monitoring the dynamic change of the H_2_^17^O water that is metabolized from ^17^O-labeled O_2_ gas (Mateescu et al., 1989; Pekar et al., 1991; Fiat and Kang, 1992, 1993; Reddy et al., 1996; Arai et al., 1998; Ronen et al., 1998; Zhu et al., 2002; Zhang et al., 2004; Atkinson and Thulborn, 2010; Kurzhunov et al., 2017; Niesporek et al., 2018). Generally, *in vivo* ^17^O-MR imaging method shares a similar principle as the well-established ^15^O-PET technique (Lenzi et al., 1981; Mintun et al., 1984) for imaging CMRO_2_. Both modalities apply isotope-labeled oxygen gas inhalation in the measurement: ^17^O_2_ for ^17^O-MR and ^15^O_2_ for ^15^O-PET. After the inhalation, the isotope-labeled O_2_ molecules bind to hemoglobin during the gas exchange in the lung and are subsequently delivered to the brain cells through blood circulation, perfusion and diffusion, and reduced by the cytochrome oxidase in the mitochondria to form the isotope-labeled water. One labeled oxygen molecule produces two labeled water molecules in the mitochondria, which can be washed out from the brain cells, enter the venous system and back to the heart via blood circulation.

**Figure 3:**
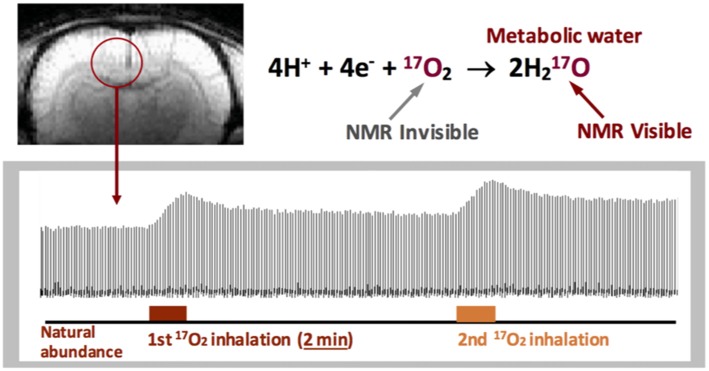
Stack plots of rat brain tissue H_2_^17^O spectra obtained from a single voxel selected from the 3D ^17^O MRSI datasets with two consecutive ^17^O_2_ inhalations (~2 min each) for repeated CMRO_2_ and cerebral blood flow (CBF) imaging measurements. ^17^O_2_ in either blood or brain tissue is NMR invisible. Figure adapted from Zhu et al. (2007).

Despite the common principle, there are fundamental differences between the ^17^O-MR and ^15^O-PET techniques in imaging CMRO_2_. ^15^O-PET cannot distinguish the radioactive signals attributed from the metabolic substrate (^15^O_2_) and the metabolic product (H_2_^15^O). Therefore, a standard PET-based CMRO_2_ imaging method requires a complicate CMRO_2_ quantification model plus multiple measurement procedures with: (i) inhalation of ^15^O_2_ gas; (ii) injection of H_2_^15^O tracer; and (iii) inhalation of C^15^O gas (Mintun et al., 1984), which substantially increase the total scanning time, radioactive dose and the measurement cost. The *in vivo* ^17^O MR imaging method, on the other hand, only detects the metabolically generated and isotope-labeled H_2_^17^O. ^17^O_2_ molecules, either freely dissolved or bound to hemoglobin are “invisible” to the *in vivo* ^17^O detection (Figure 3) owing to the extremely broad ^17^O resonance linewidth (Zhu et al., 2005; Zhu and Chen, 2011). This feature greatly simplifies the ^17^O-MR based CMRO_2_ imaging measurement that uses a non-radioactive and stable isotope, and thus, is more safer for human application (Zhang et al., 2004; Zhu et al., 2005; Atkinson and Thulborn, 2010).

The dynamics of the ^17^O MR signal from the brain tissue H_2_^17^O measured during and after an ^17^O_2_ inhalation reflects an interplay of three physiological processes: (i) oxygen consumption to produce labeled H_2_^17^O in the mitochondria, (ii) washout of labeled H_2_^17^O from the brain cells via blood circulation, and (iii) “recirculation” of labeled H_2_^17^O generated in the body re-entering the brain. The mass balance equation accounted the contributions from all three processes can be used for CMRO_2_ quantification (Pekar et al., 1991; Zhu et al., 2002, 2005; Zhang et al., 2004; Atkinson and Thulborn, 2010):

(1) $$\frac{dC_{b}\left(t\right)}{dt}=2\cdot \alpha \left(t\right)CMRO_{2}+CBF\cdot \left[C_{a}\left(t\right)-\frac{C_{b}\left(t\right)}{\lambda }\right]$$

where C_a_(t), and C_b_(t) are the time-dependent and ^17^O-isotope labeled H_2_^17^O concentration in the arterial blood and brain tissue, respectively; α(t) is the ^17^O enrichment fraction of the blood-contained ^17^O_2_; λ is the brain/blood partition coefficient; the factor of 2 in Equation 1 accounts for the production of two H_2_^17^O molecules from one ^17^O_2_ molecule (Zhu et al., 2002; Zhang et al., 2004).

As demonstrated in Figure 3, there are three distinct phases in the brain H_2_^17^O time course covering the baseline, inhalation and post-inhalation periods (Zhu et al., 2002). The signals in the first phase representing the natural abundance H_2_^17^O in the brain tissue can serve as an internal reference for quantifying the brain H_2_^17^O concentration and its change during the second and third phases. Equation 1 can be employed to calculate the CMRO_2_ and CBF values, and estimate OEF (detailed quantification modeling and simplified approaches can be found in the literature (Zhu et al., 2002, 2013a,b).

One attractive feature of the ^17^O-MR based CMRO_2_ imaging approach is it enables repeated CMRO_2_ measurements since the metabolized H_2_^17^O signal in the brain can reach a new steady-state within a short time (e.g., <10 min in rodents, see Figure 3) at the end of the ^17^O_2_ inhalation, so subsequent CMRO_2_ measurements can be performed in the same subject within the same imaging session (Zhu et al., 2007). This capability is important for studying CMRO_2_, CBF and OEF and their changes due to physiopathological perturbations where multiple measurements under different conditions are required.

**Figure 4:**
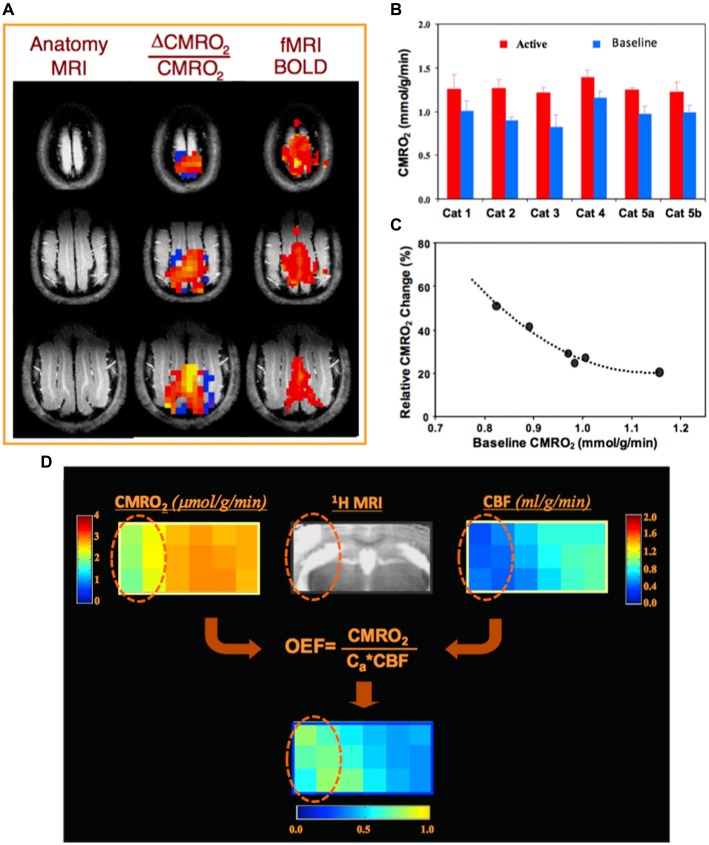
**(A)** Anatomic brain images (left), fMRI blood oxygenation level dependent (BOLD) maps (right) and corresponding 3D functional CMRO_2_ activation maps (middle) obtained from three representative image slices in a cat visual cortex, showing a significant CMRO_2_ increase during visual stimulation. **(B)** Individual CMRO_2_ values measured from the activated cat visual cortex region during visual stimulation as well as at control condition (baseline). Two functional studies conducted at different days in Cat 5, showing excellent CMRO_2_ imaging reproducibility. **(C)** A strong correlation of baseline CMRO_2_ level and activated CMRO_2_ change among individual animals. Figure adapted from Zhu et al. (2009). **(D)** *In vivo* ^17^O MRS imaging from a representative image slice in a mouse with middle cerebral artery occlusion (MCAO) preparation, showing significant reductions of CMRO_2_ and CBF, and an elevated oxygen extraction fraction (OEF) in the ischemic brain region (cycled) affected by MCAO as compared to the intact tissue in the contralateral hemisphere. Figure adapted from Zhu et al. (2013a) with permission of Elsevier Inc.

For example, Figure 4A illustrates a functional study of blood oxygenation level dependent (BOLD) contrast and CMRO_2_ changes in cat brain during visual stimulation (Zhu et al., 2009). Two 3D ^17^O CMRO_2_ imaging measurements, with and without visual stimulation, were performed on each animal. A significant increase in CMRO_2_ (~30%) was detected in the activated visual cortical regions (Figures 4A,B); and interestingly, a strong inverse relation between the baseline CMRO_2_ level and stimuli-induced CMRO_2_ relative change across different subjects (Figure 4C) was observed (Zhu et al., 2009). Figure 4D demonstrates a preclinical application of the quantitative ^17^O-MR imaging methodology for simultaneous and completely noninvasive mapping of CMRO_2_, CBF and OEF in mouse brain using a brief ^17^O_2_ inhalation (2–3 min), showing impaired CMRO_2_ and CBF and elevated OEF in the ischemic brain region as compared to the intact brain tissue in the contralateral hemisphere (Zhu et al., 2013a).

For human brain application, due to the large body size, slow blood circulation and exchange of ^17^O labeled and non-labeled oxygen gas in human lung, it is more challenging to reliably quantify CMRO_2_, and a more sophisticated CMRO_2_ quantification model is required (Atkinson and Thulborn, 2010; Zhu et al., 2014). Recently, we have demonstrated the feasibility for noninvasively imaging all three parameters of CMRO_2_, CBF and OEF using a brief (2–3 min) ^17^O_2_ inhalation in human visual cortex under resting condition and their changes in response to visual stimulation (Zhu et al., 2014).

## Studying Cerebral ATP Energy Metabolism and NAD Redox Using *in vivo* ^31^P MRS Technique

**Figure 5:**
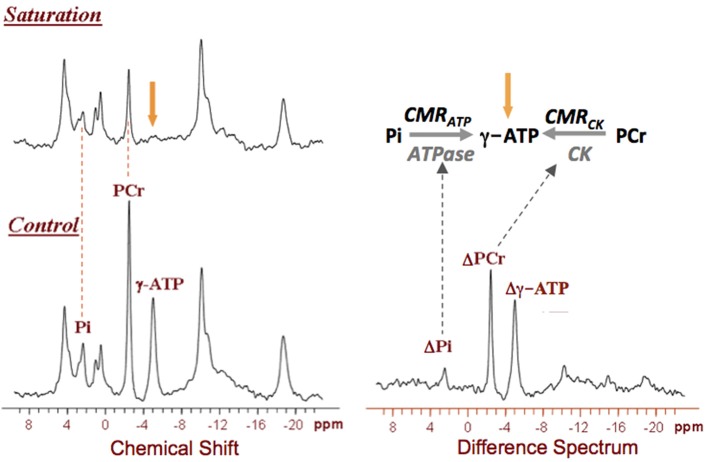
*In vivo* ^31^P spectra obtained from a representative healthy human brain in the absence (control) and presence of γ-ATP resonance saturation at 7T, and their difference spectrum. The signal reductions in the Pi and Phosphocreatine (PCr) resonances can be used to calculate the values of CMR_ATP_ and CMR_CK_, respectively. Figure adapted from Lei et al. (2003a).

*In vivo* ^31^P MRS is a powerful tool for studying cerebral phosphorus metabolism and neuroenergetics without the need for any isotopically labeled substrate. It not only detects various phosphorus metabolites, but also determines intracellular pH and free Mg^2+^ concentration of the brain tissue (Ackerman et al., 1980; Hetherington et al., 2002; Lei et al., 2003b; Du et al., 2007, 2008; Zhu et al., 2012). Furthermore, when it combines with the magnetization transfer (MT) preparation (^31^P MRS-MT), the enzyme activities and metabolic fluxes via the F_1_F_0_-ATPase and CK reactions can be measured and quantified (Frosén and Hoffman, 1963; Shoubridge et al., 1982; Uǧurbil, 1985; Lei et al., 2003a; Du et al., 2007; Ren et al., 2017). Therefore, the *in vivo* ^31^P MRS-MT technique can be used to noninvasively study abnormal mitochondrial function associated with energetic impairment in neurodegenerative diseases such as AD (Schägger and Ohm, 1995). Figure 5 displays a typical ^31^P MRS-MT dataset obtained in human brain at 7T. The signal reductions of the PCr and Pi resonances in the presence of γ-ATP saturation as compared to that of control can be used to calculate the “forward” metabolic fluxes for the CK reaction (i.e., PCr→ATP) and ATPase reaction (i.e., Pi→ATP), i.e., CMR_CK_ and CMR_ATP_, respectively (Lei et al., 2003a; Du et al., 2007).

**Figure 6:**
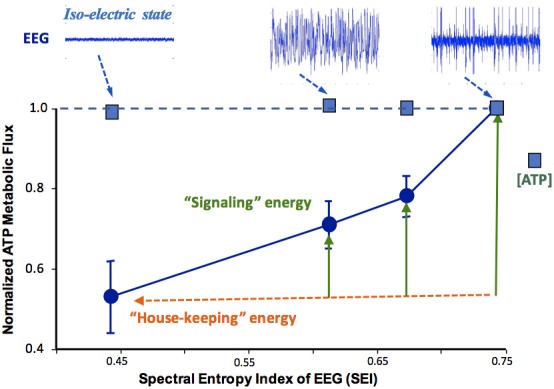
Relationship of the rat brain electroencephalogram (EEG) activity level (top tracers) and normalized CMR_ATP_ or cerebral ATP concentration determined under different brain states. The EEG signal was quantified by the spectral entropy index (SEI). The CMR_ATP_ value correlates strongly with SEI, while intracellular ATP concentration remains constant even at the iso-electric state. Figure adapted from Du et al. (2008).

As shown in Figure 6, the *in vivo* ^31^P MRS-MT method can be used to investigate the relation between the neuronal activity level and the ATP production rates at different brain states (Du et al., 2008). In this study, a strong positive correlation between the spontaneous brain electroencephalogram (EEG) activity and CMR_ATP_ was reported (Du et al., 2008); it was also found that when all electrophysiological signals are stopped (i.e., in an isoelectric state), the brain still consumes a significant portion of ATP energy for “house-keeping” and maintaining the cellular integrity; and the brain ATP concentration remains constant while CMR_ATP_ could vary ~50% over a wide range of neuronal activity levels (see Figure 6). These findings suggest that under physiological conditions, the cerebral energy metabolism is effectively regulated to maintain the intracellular ATP homeostasis; and the metabolic rate of CMR_ATP_ should be a better biomarker for assessing energetic state of healthy brains (Du et al., 2008).

With improved sensitivity and spectral resolution at ultrahigh field of 7T and advancement in developing UHF radiofrequency (RF) coils, *in vivo* ^31^P MRS-MT approach can be combined with 3D chemical shift imaging (CSI) to map the ATP metabolic rates in human brain with whole-head coverage. This makes it possible to differentiate CMR_ATP_ and CMR_CK_ between the human brain gray matter (GM) and white matter (WM), which led to the finding of three times higher CMR_ATP_ and CMR_CK_ in GM than WM. Also, it has been found that on average, a single neuron consumes ~4.7 billion ATP molecules per second in human cortex at resting condition based on the direct CMR_ATP_ imaging measurement (Zhu et al., 2012).

**Figure 7:**
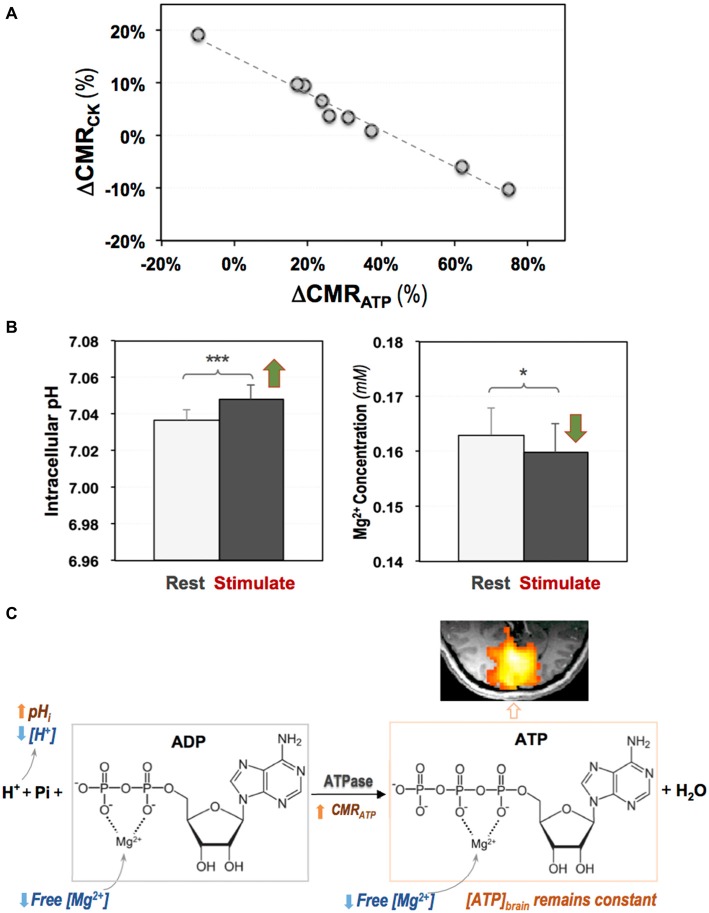
**(A)** A strong and negative correlation between the stimulus-evoked CMR_CK_ and CMR_ATP_ changes among individual subjects; and **(B)** increased intracellular pH and decreased intracellular free Mg^2+^ level in human visual cortex during visual stimulation. **(C)** Schematic illustration of complex and coherent changes in ATPase activity, ATP production rate (CMR_ATP_), intracellular pH and free (Mg^2+^) in response to brain stimulation (Zhu et al., 2018). Two-tailed paired *t*-test indicating significant differences detected comparing the two conditions with *p < 0.05 and ***p < 0.001.

Given the high ATP expenditure in a resting human brain, how a brain at working-state fulfills its energetic requirement is an important question for understanding the fundamental role of cerebral energetics in brain function and health. By applying the 3D ^31^P CSI-MT imaging technique in human visual cortex at 7T, the regional CMR_ATP_ and CMR_CK_ at rest and during visual stimulation were directly measured; and a significant stimulus-induced and highly correlated neuroenergetic changes was detected, indicating that the ATPase and CK reactions play distinctive and complementary roles in supporting evoked neuronal activity and maintaining the intracellular ATP homeostasis (Zhu et al., 2018). Figure 7 summarizes the results of this study showing a strong and negative correlation between the task-evoked CMR_ATP_ and CMR_CK_ changes in the activated human visual cortex among individual subjects (Figure 7A), and a significant increase in the intracellular pH accompanied by a reduction in the intracellular free [Mg^2+^] during the visual stimulation (Figure 7B). The findings of this original study provide interesting new insights into the mechanism of brain ATP energy metabolism and regulation in supporting evoked neuronal activity (Figure 7C), and demonstrate that the *in vivo* ^31^P-MT imaging technique is a sensitive and highly valuable neuroimaging tool for quantitatively studying energy metabolism in human brain.

Brain energy metabolism and regulation are controlled by the metabolic coenzyme NAD and its redox state presented by the parameter of RX_NAD_ (= [NAD^+^]/[NADH]). Extensive biological and cellular studies indicate that NAD^+^ also functions as a co-substrate for several important enzymes including Sirtuins, poly-ADP-ribose polymerases (PARPs) and CD38/157 that play critical roles in cellular signaling, cell death, aging and longevity. Intracellular NAD^+^ depletion has emerged as an indicator of aging and neurodegeneration, and thus, it is considered as a new therapeutic target for aging-related disorders and neurodegenerative diseases (Ying, 2007; Mouchiroud et al., 2013; Imai and Guarente, 2014; Verdin, 2015; Guarente, 2016; Mills et al., 2016; Schultz and Sinclair, 2016; Fang et al., 2017).

**Figure 8:**
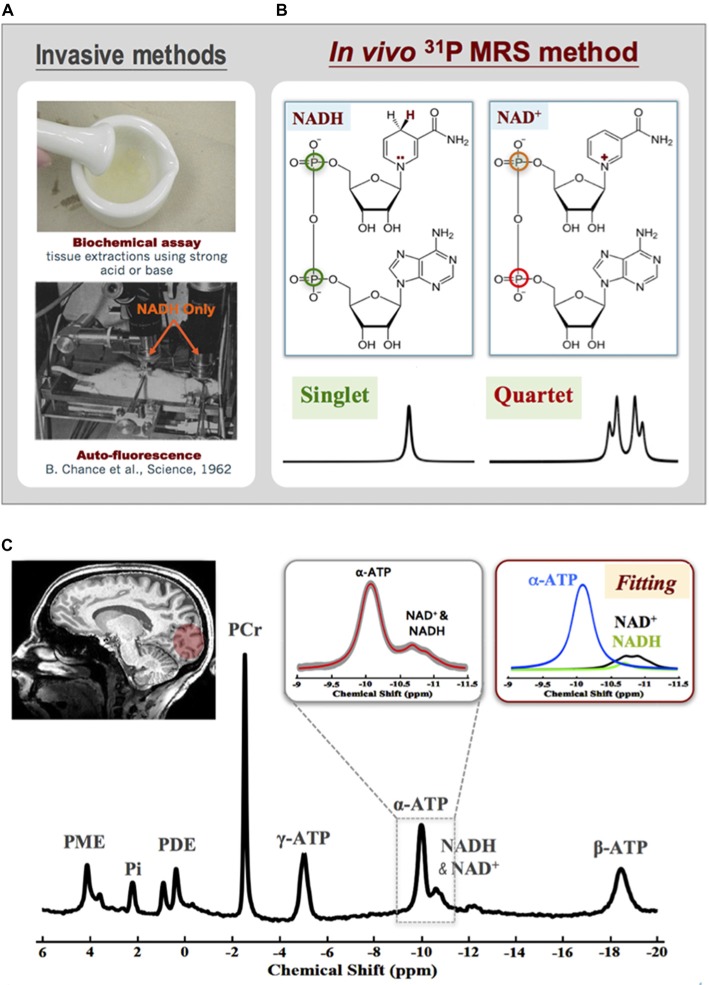
**(A)** Two invasive methods are available for assessing intracellular NAD contents based on biochemical assay (top) or auto-fluorescence technique. **(B)** Molecular structures of NAD^+^ and NADH and their distinct ^31^P spectral patterns (singlet for NADH and quartet for NAD^+^). **(C)** A representative ^31^P spectrum obtained from the occipital lobe of a healthy subject at 7T. The expanded spectra in the inserts display a chemical shift range of −9.0 to −11.5 ppm with original ^31^P signal (gray trace) and model fitted spectrum (red trace) of α-ATP and NAD. The individual fitting components of γ-ATP (blue), NAD^+^ (black) and NADH (green) are also showed in the top-right panel. Figure adapted from Zhu et al. (2015b).

Despite the crucial roles of NAD in cellular energy metabolism and signaling, determining intracellular NAD contents and redox state is difficult, especially in live brains. Only two invasive methods are available: one is the biochemical assay (Zhang et al., 2006; Yang et al., 2007; Xie et al., 2009) and the other relies on the auto-fluorescence signal of the NADH but not NAD^+^ (Chance et al., 1962; see Figure 8A). Few years ago, an *in vivo* ^31^P MRS-based NAD assay was developed in our laboratory that enables noninvasive assessment of NAD^+^ and NADH contents and RX_NAD_ in animal and human brains (Lu et al., 2014b, 2016a; Zhu et al., 2015b). This new method utilizes a theoretical NMR spectral model to describe the ^31^P resonances of NAD^+^ and NADH and their spectral patterns at a given magnetic field strength. As shown in Figure 8B, the molecular structure of NAD^+^ only differs from NADH by one H^+^ and two electrons. This subtle structural difference makes the shielding environment of the phosphorus spins in the NAD^+^ molecule (two different ^31^P spins) substantially different from that of NADH (two identical ^31^P spins). Based on the NMR theory, the second-order coupling effect applies to the two-spin system of NAD^+^, leading to a well-defined quartet of resonances with the signal intensity ratios and chemical shifts varying with the field strength; conversely, the NADH displays a single resonance with doubled intensity. The spectral patterns of NAD^+^ quartet and NADH singlet, therefore, can be precisely predicted at any given field strength using a quantification model that describes all ^31^P signals of NAD^+^, NADH and α-ATP. After least-square fitting of the *in vivo* ^31^P spectrum, the values of [NAD^+^], [NADH] and RX_NAD_ can be calculated using the α-ATP signal as an internal concentration reference (Lu et al., 2014b). The newly developed *in vivo* NAD assay has been applied to the healthy human at 7T. Excellent SNR and spectral quality as shown in Figure 8C ensures the reliable fitting of NAD^+^, NADH and α-ATP resonances and the quantification of [NAD^+^] (≈0.30 ± 0.02 mM), [NADH] (≈0.06 ± 0.01 mM) and RX_NAD_ (= 4.8 ± 0.9) in the healthy human brain (Zhu et al., 2015b).

**Figure 9:**
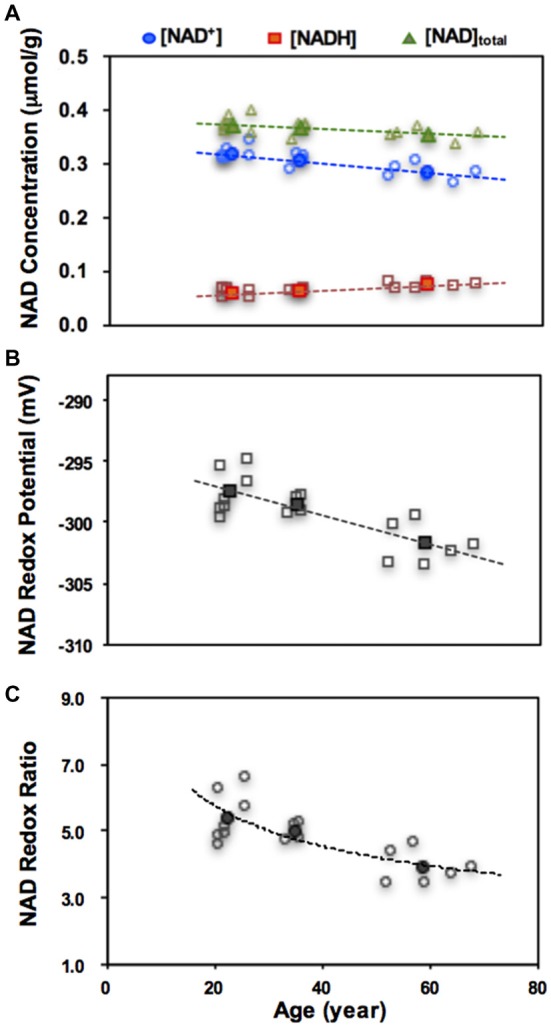
Age dependences of intracellular NAD^+^, NADH, total NAD concentrations **(A)**, the NAD redox potential **(B)** or redox ratio **(C)** observed in healthy human brains. The open symbols represent data of individual subjects and the filled symbols are the average data from three age groups of young (21–26 year, *n* = 7), middle (33–36 year, *n* = 4) and old (59–68 year, *n* = 6) subjects. Figure adapted from Zhu et al. (2015b).

A growing number of evidence suggests a close link between the abnormal brain NAD^+^ and amyloid beta-peptide in AD (Wu et al., 2014), and therapies that aim to restore intracellular NAD^+^ level have shown promise for repairing the DNA damage or protecting against age-related cellular damage (Braidy et al., 2008, 2011). The ^31^P MRS-based *in vivo* NAD assay provides an ideal tool to monitor the NAD changes in the human brain. Figure 9 shows an application of the NAD assay in healthy subjects, which detected strong age-dependent changes in [NAD^+^], [NADH], [NAD]_total_ (=[NAD^+^]+[NADH]) and RX_NAD_ (Zhu et al., 2015b). A decrease in the NAD^+^/NADH redox ratio in normal human brain indicates that the glucose-oxygen metabolic balance is shifted toward a slower mitochondrial oxidative phosphorylation, leading to a lack of ATP production capacity in the aging brain.

**Figure 10:**
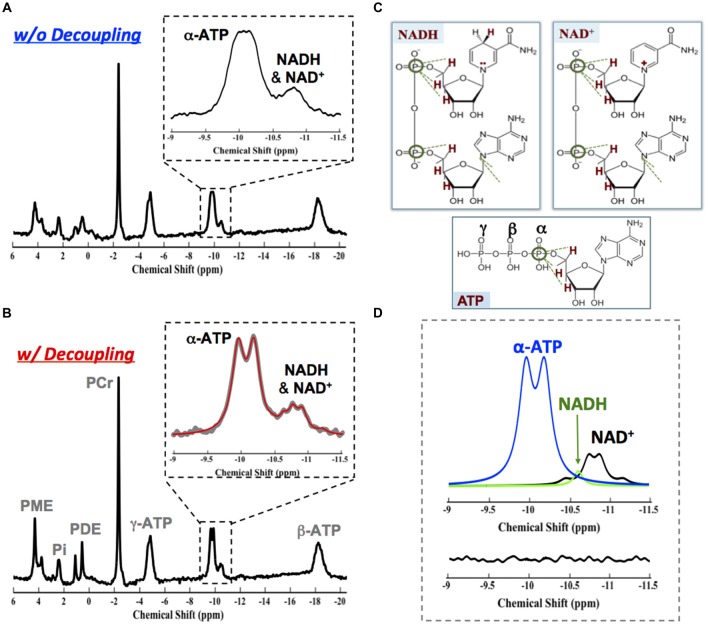
*In vivo* ^31^P brain spectrum collected in the absence **(A)** and presence **(B)** of ^1^H decoupling on the water resonance from a representative healthy subject at 4T. The ^1^H decoupling significantly reduces the linewidth of γ-ATP, NAD^+^ and NADH resonances owing to the close proximity between their phosphorus spins to the protons as shown in **(C)**. **(D)** Fitting results showing individual components of γ-ATP (blue), NAD^+^ (black) and NADH (green) signals and a small residue. Figure adapted from Lu et al. (2016a).

Interestingly, we have reported that the *in vivo* ^31^P MRS NAD assay could also be employed at relatively lower field. Similar performance at 7T can be achieved at 4T with incorporation of ^1^H decoupling into the ^31^P NAD assay (Zhu et al., 2015b; Lu et al., 2016a). The advanced NAD assay approach at 4T significantly improves the spectral resolution and the SNR of the NAD^+^, NADH and α-ATP (Figures 10A,B) owing to the proximity of the nearby protons (Figure 10C); excellent model fittings (Figure 10D) and identical RX_NAD_ value (5.3 ± 0.4, *N* = 7, age: 23 ± 4 years) as that of 7T (5.4 ± 0.8, *N* = 7, age 23 ± 2 years) were obtained (Zhu et al., 2015b; Lu et al., 2016a). This result confirms the potential of *in vivo* NAD assay for translational applications at the field strength of clinical scanners, for instance, at 3T.

In summary, the advanced *in vivo* X-nuclear MRS imaging techniques as reviewed in this article can provide quantitative measures of key physiological parameters representing metabolite contents, tissue properties and metabolic rates involving major energetic pathways in live brains. The multi-nuclear MRS imaging measurements can benefit substantially from the high/ultrahigh magnetic field for improving sensitivity and reliability, and thus, these valuable metabolic imaging tools can be used to noninvasively assess the brain energetic changes in aging and neurodegenerative diseases. Although the neuroenergetic measurements and the quantitative markers described herein have shown feasibility and great potential in early detection of abnormal cerebral metabolism related to aging and neurodegeneration, their use in clinical practice still requires time and more efforts; nevertheless, the FDA approval of the 7T human scanner for clinical diagnosis of brain diseases will speed up the process. The same imaging methods are also suitable for studying the physiological functions and aging dependence of other organs such as the heart and skeletal muscle.

## Author Contributions

X-HZ and WC made equal contribution for writing and editing this review article.

## Conflict of Interest Statement

The authors declare that the research was conducted in the absence of any commercial or financial relationships that could be construed as a potential conflict of interest.
